# Spatial metaphors and the design of everyday things

**DOI:** 10.3389/fpsyg.2022.1019957

**Published:** 2022-11-21

**Authors:** Benjamin Pitt, Daniel Casasanto

**Affiliations:** ^1^Department of Psychology, University of California, Berkeley, Berkeley, CA, United States; ^2^Department of Psychology, Cornell University, Ithaca, NY, United States

**Keywords:** metaphor, design, space, time, number, emotion, mappings, human-computer interaction

## Abstract

People use space (e.g., left–right, up-down) to think about a variety of non-spatial concepts like time, number, similarity, and emotional valence. These spatial metaphors can be used to inform the design of user interfaces, which visualize many of these concepts in space. Traditionally, researchers have relied on patterns in language to discover habits of metaphorical thinking. However, advances in cognitive science have revealed that many spatial metaphors remain unspoken, shaping people’s preferences, memories, and actions independent of language – and even in contradiction to language. Here we argue that cognitive science can impact our everyday lives by informing the design of physical and digital objects *via* the spatial metaphors in people’s minds. We propose a simple principle for predicting which spatial metaphors organize people’s non-spatial concepts based on the structure of their linguistic, cultural, and bodily experiences. By leveraging the latent metaphorical structure of people’s minds, we can design objects and interfaces that help people think.

## Introduction

People are spatial thinkers. From early in life, humans form implicit associations between space (e.g., length, size, position) and a variety of non-spatial conceptual domains, including time, number, and emotional valence (Casasanto and Henetz, 2012; Fischer and Shaki, 2014; de Hevia et al., 2017; Starr and Srinivasan, 2021). By adulthood, people in many cultures use spatial metaphors to recall the past and plan the future, to perform mental calculations, and to evaluate competing options. Although these spatial metaphors are typically unconscious, they shape people’s preferences, memories, and actions, and are often reflected in cultural artifacts and practices. For example, the mental number line – an implicit association between space and number – is physically manifested in a variety of cultural artifacts, like the number lines that appear on kindergarten walls, in charts and graphs, and on computer keyboards. Other spatial metaphors underly the intuition that, to show approval, social media users should give a “thumbs up,” not a thumbs down, and that to go “back” to a previous webpage, browser users should press the arrow pointing left, not the arrow pointing right.

Psychologists and user experience (UX) designers have long appreciated the role of space in human cognition (e.g., Lakoff and Johnson, 1980; Norman, 1988; Tversky et al., 1991; Casasanto, 2010), and have typically relied on patterns in language to identify patterns in thinking. However, in the first decades of the 21^st^ century, cognitive scientists have shown that the spatial metaphors shaping people’s minds are not faithfully reflected in language (Casasanto and Bottini, 2014a). Rather, people’s implicit spatial metaphors are shaped by the specifics of their spatial experiences, and may vary independent of metaphors in their language. Here we discuss the dynamics and development of spatial metaphors, and how they can be leveraged in the design of products, environments, and user interfaces.

## What is a spatial metaphor?

In the context of human-computer interaction, the word “metaphor” is most often used to describe the desktop, menu, and folder metaphors (among others) that have become ubiquitous elements of the graphical user interface. No doubt, these metaphors have been wildly successful in making computers accessible to a broad audience, allowing users to apply what they already know about physical objects (like an actual file folder) to digital interfaces (see Carroll et al., 1988; Marcus, 1998; Hurtienne and Israel, 2007). However, these analogical metaphors are far from the only metaphors that influence the way people think or interact with user interfaces (UIs). A different kind of metaphor – based on our interactions with the spatial environment – are found in the minds of all people from early in life, and these implicit *spatial metaphors* shape the way we think, feel, and act in predictable ways. Unlike analogical metaphors like *desktop* and *folder*, spatial metaphors require no experience with any particular object (like a desk) and are not limited to a single UI element (like a desktop). Rather, spatial metaphors can be learned from a wide variety of experiences and are broadly applicable across contexts, as they structure some of our most fundamental concepts, including our concepts of time, quantity, similarity, good, and evil.

In some cases, spatial metaphors are reflected in language. For example, when we say *His spirits were soaring* or *She is feeling low*, we are invoking a spatial metaphor linking “up” with positive emotional valence and “down” with negative emotional valence (Lakoff and Johnson, 1980): the same implicit spatial metaphor that gives rise to the conventionalized thumbs up gesture (and the corresponding “Like” button). Dozens of these spatial metaphors are hiding in plain sight throughout language, linking numerous non-spatial conceptual domains with various aspects of space on all three axes (i.e., lateral, vertical, and sagittal), and across dimensions (i.e., 1-D, 2-D, or 3-D). For example, in English, we use vertical space to talk about high and low numbers, lateral space to talk about the left and right poles of the political spectrum, and sagittal space to talk about moving meetings forward or back in time. Quantities can be big or small; vacations can be long or short; acquaintances can be close or distant.

According to Conceptual Metaphor Theory (Lakoff and Johnson, 1980), these metaphors in language are more than just figures of speech; rather, they reflect the fact that “most of our ordinary conceptual system is metaphorical in nature” (p. 454). On this proposal, people use mental representations in a *source* domain (like space) to scaffold their representations in a *target* domain (like time, number, or emotional valence), which is typically more abstract. Using the source domain to conceptualize the target domain (e.g., using space to think about time) is hypothesized to support people’s ability to make inferences in the target domain. A growing body of experimental evidence supports the proposal that in addition to talking metaphorically about abstract domains like time, number, and similarity, people also *conceptualize* these domains metaphorically, often using space as the source domain – that is, people use space for thinking (for a review, see Casasanto and Bottini, 2014a).

Given that such spatial metaphors (i.e., mental metaphors whose source domain is space) help people think, they provide a valuable starting point for designing UIs; users bring to any interface a set of implicit biases about the way(s) in which many abstract domains should be spatialized. By designing interactions that are congruent with people’s implicit spatial metaphors, designers can leverage the latent structure of users’ minds. By contrast, violating those metaphors (or just ignoring them) results in user experiences that may be unintuitive, unpleasant, or even dangerous. The better we understand the metaphorical structure of people’s minds, the better we are able to design experiences that leverage that structure.

Although metaphor theorists have typically treated metaphorical language and metaphorical thinking as if they were inseparable, there are many well-established dissociations between metaphors in language and in the mind. People’s thoughts are structured metaphorically in ways that do not appear in language at all and, in some cases, may contradict patterns in language. Therefore, to fully understand the metaphorical structure of people’s minds, we must look beyond language. Whereas metaphor theorists have traditionally used a single term (i.e., “conceptual metaphor”) to refer to both patterns in language and patterns in thinking, here we will use two terms to make this critical distinction; *linguistic metaphor* refers to metaphorical structures in language and *mental metaphor* refers to metaphorical structures in thinking (Casasanto, 2008).

Below, we will begin by discussing two mental metaphors that have corresponding linguistic metaphors in English (i.e., “Spatial metaphors in language and thought”) and highlight their implications for design. We will then turn to mental metaphors that have no corresponding linguistic metaphors (i.e., “Unspoken spatial metaphors”) and illustrate how, despite their absence from language, these metaphors have potent cognitive and behavioral effects. In subsequent sections, we will discuss how (and why) spatial metaphors vary across cultures and across individuals, and outline a simple principle for anticipating the particularities of a given person’s spatial metaphors, whether or not those metaphors are reflected in language.

## Spatial metaphors in language and thought

### Good is up

Some mental metaphors are reflected in language. The Like button, mentioned above, reflects an association between vertical space and emotional valence (i.e., positive and negative) that is commonplace in many languages. The GOOD IS UP metaphor also runs deeper than language (e.g., Meier and Robinson, 2004; Havas et al., 2007), and activating this metaphor — *via* interaction with the physical environment — can shape the way we feel, how we act, and what we remember. For example, moving objects upward (i.e., from a low container to a higher container) caused people in one study to recall autobiographical memories with positive emotional valence, whereas moving objects downward caused them to recall more negative memories (Casasanto and Dijkstra, 2010). Even illusory vertical motion (induced by visual gratings) can have these direction-specific effects, modulating the emotional content of their recollections and their self-reported mood (Seno et al., 2013).

Beyond partly determining what people recall, the GOOD IS UP metaphor can also influence how well we learn. In another experiment, people studied the meanings of “alien” words using flashcards that they placed into an upper or lower bin, according to a rule that was either congruent with the GOOD IS UP metaphor or incongruent with it (Casasanto and de Bruin, 2019). People who placed the positive words in the upper bin and the negative words in the lower bin (congruent with the GOOD IS UP metaphor) showed a significant improvement in vocabulary learning compared to baseline, whereas those who placed their cards the other way (incongruent with the implicit spatial metaphor) showed a significant decrement. Leveraging this implicit spatial metaphor improved learning, and violating it impaired learning. This finding demonstrates what is at stake in respecting people’s mental metaphors. The question is not simply whether designers do or do not use these metaphors to improve people’s experience. Rather, the question in many cases is whether these metaphors are used intentionally to improve people’s thinking, or (unintentionally) to impair it.

### Good is right

In addition to the GOOD IS UP metaphor, most people also have a GOOD IS RIGHT metaphor linking negative emotions with the left side of space and positive emotions with the right (Casasanto, 2009).^1^ This metaphor has subtle but systematic correlates in English: Being dexterous (i.e., right) is good but being sinister (i.e., left) is bad; likewise, a trusted colleague is a “right-hand man,” whereas a clumsy dancer has “two left feet.” Yet, despite these clues in language, links between lateral space and valence remained largely unknown to cognitive science even decades after Lakoff and Johnson (1980) wrote about links between vertical space and valence (i.e., GOOD IS UP). In fact, the left–right axis was long thought to be “neutral” in valence (Tversky, 2001, p 101; see also Clark, 1973). Dozens of studies have now shown that most people implicitly associate good things with the right side of space and bad things with the left (e.g., Pitt and Casasanto, 2018).

The lateral space-valence metaphor has widespread effects on the way people perceive, remember, and act, with clear implications for the design of UIs. Encountering objects on the right or left side of space influences how people *feel* about them. Position in lateral space matters when choosing which of two products to buy, which of two job applicants to hire, which of two alien creatures looks more honest (Casasanto, 2009), or which person to date or to befriend (Zhao et al., 2018). Most people are biased to choose the person, product, or creature they find on the right of the page or the computer screen. This spatial metaphor can also color our memories; when asked to remember the location of fictitious events, most people err to the right (and upwards) for positive events and to the left (and downwards) for negative events (Brunyé et al., 2012). When asked to arrange emotional faces on a left–right continuum, most people are biased to place the unhappy faces on the left and the happy faces on the right (Freddi et al., 2016).

**Figure 1:**
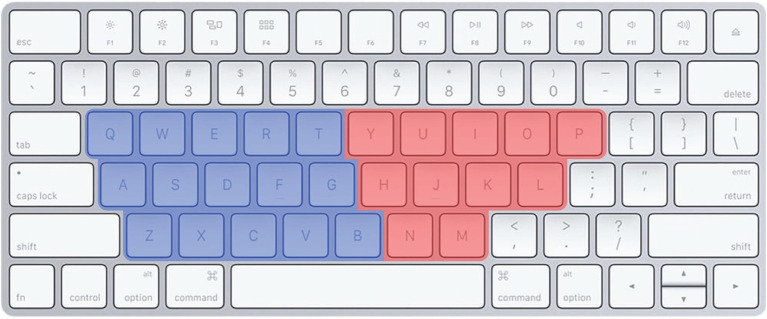
On the QWERTY keyboard, some letters are typed with the left hand (blue) and some are typed with the right hand (red). The left–right distribution of letters on this interface influences the meaning of words as predicted by the GOOD IS RIGHT metaphor. Image by Fletcher, distributed under a CC-BY 4.0 license.

The GOOD IS RIGHT metaphor shapes our minds in some surprising ways. Notably, this mental metaphor appears to have unintended effects on language *via* one of the most common UIs in the industrialized world: the QWERTY computer keyboard. On a standard QWERTY keyboard, some of the letters are typed on the left side with the left hand (Figure 1, blue keys) and some are typed on the right side with the right hand (Figure 1, red keys). Given that most people associate bad with left and good with right, the layout of the keys could influence the emotional valence of the letters, and therefore of any typed word. This relationship between the meanings of words and the way they are typed is now known as the “QWERTY effect” (Jasmin and Casasanto, 2012) and has been found in a variety of contexts and languages. In a first test of this effect, the emotional valence of words (as rated by English speakers) was predicted in part by the way they were typed on the keyboards used to produce English, Dutch, and Spanish; words (and pseudowords) with more right-side letters were found to be, on average, more positive in meaning. This effect was later extended to Portuguese and German words, and found for ratings of single letters, *per se*: Letters farther to the right on the keyboard were rated to be more positive on average (Casasanto et al., 2014).

Not only does QWERTY subtly shape the meanings of words, it also predicts which words people choose in natural language to describe products, experiences, and even people. In a corpus study of more than a dozen major websites, people reliably used more right-sided words to describe the products (e.g., on Amazon.com), videos (e.g., on YouTube.com), and businesses (e.g., on Yelp.com) that they rated more positively (e.g., 1–5 stars; Garcia and Strohmaier, 2016). Likewise, the proportion of right-side letters in the name of a given product, video, or businesses predicted how well it was rated; those with names typed on the right got higher ratings than those with names typed on the left (*ibid*). QWERTY also appears to influence how people name their children. Since the widespread adoption of the QWERTY keyboard, names with more right-side letters have dramatically increased in popularity while names with more left-side letters have decreased in popularity in the US. Likewise, names invented since the adoption of QWERTY have a decidedly right-sided bias, comprising more right-side letters compared to names in use before QWERTY (Casasanto et al., 2014). The QWERTY effect illustrates one way in which the interfaces we use to produce language can shape language in return, *via* the spatial metaphors in our minds. Although the influence of a spatial metaphor like GOOD IS RIGHT on any one word or any one experience may be small, its cumulative effect across many thousands of instances may be substantial.

## Unspoken spatial metaphors

Whereas some spatial metaphors are evident in language (e.g., GOOD IS UP, GOOD IS RIGHT), others are entirely absent from language. One such metaphor is revealed by a simple exercise: If asked how best to arrange the words “today,” “tomorrow,” and “yesterday” in left–right space, many readers will find the best arrangement to be obvious: yesterday today tomorrow.

The intuition to put earlier events on the left and later events on the right is a product of our lateral *mental timeline*, a mental metaphor in which temporal succession in mapped onto left–right space (Tversky et al., 1991). People have similarly strong intuitions about the relationship between lateral space and numbers; thanks to an implicit mental number line, people associate smaller numbers with the left and larger numbers with the right (Dehaene et al., 1993; Rugani and de Hevia, 2017). Notably, these space–time and space-number metaphors are not reflected in language; numbers can be “high” or “low” but they are not “left” or “right”; events in time can be moved “forward” or “back” but not “leftward” or “rightward.”

Yet, despite their absence from language, these lateral space-number and space–time associations have robust effects on people’s behavior. For example, in tests of space-number associations, people spontaneously generate larger numbers when turning their head to the right and smaller numbers when turning to the left (Loetscher et al., 2008b). Likewise, they shift their attention to the left in response to small numbers, and to the right in response to larger numbers (Fischer et al., 2003; Loetscher et al., 2008a). And when performing simple arithmetic operations in their heads, people tend to overestimate the solution to addition problems and *under*estimate the solution to subtraction problems (i.e., the “operational momentum” effect; McCrink et al., 2007; Klein et al., 2014), as if overshooting their target on a mental number line. In tests of space–*time* associations, people spontaneously gesture to the left when describing earlier events in time and to the right for later events (at least in some cultures; Casasanto and Jasmin, 2012); When recalling items from a memorized sequence, people’s gaze shifts leftward when recalling earlier items and shifts rightward when recalling later items (Rinaldi et al., 2015). Given that these implicit mappings influence where people attend, how they move, and what they remember, any interface that spatializes number or time would be well-served to respect these mappings. Do they?

**Figure 2:**
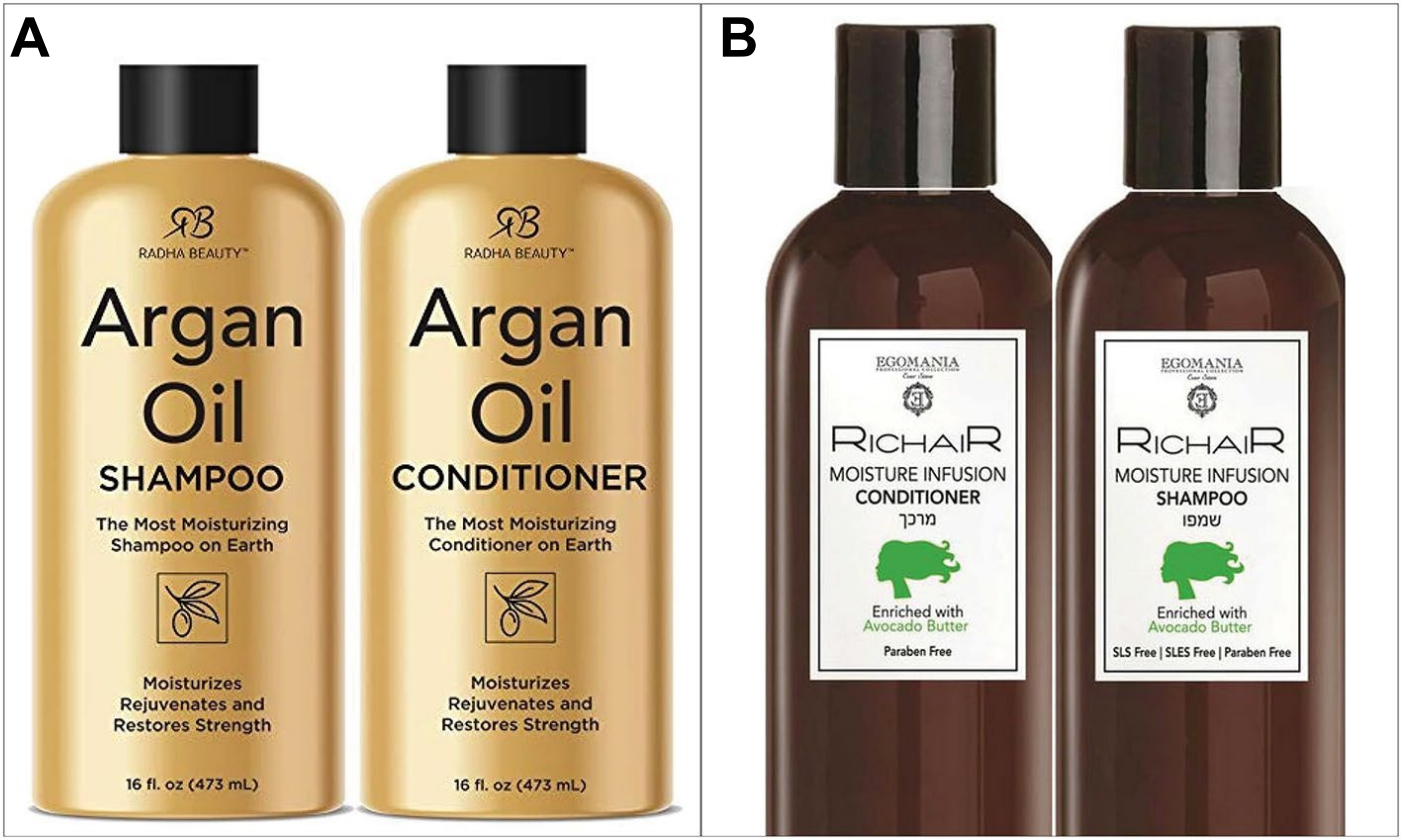
Everyday objects tend to be arranged in space according to the mental timeline, which varies in direction across cultures. **(A)** Typical arrangement of shampoo and conditioner bottles in the US, consistent with a left-to-right mental timeline. **(B)** Typical arrangement of shampoo and conditioner bottles in Israel, consistent with a right-to-left mental timeline. Reproduced with permission from RozetkaLTD, available at https://rozetka.com.ua.

Although unspoken, the specific space-number and space–time mappings just described are intuitive enough that they are incorporated into much of the built environment, digital and otherwise. The mental number line is reflected in charts and graphs, in which smaller numbers appear to the left of larger numbers, and in a variety of common interfaces, ranging from the number keys at the top of your computer keyboard to the dial on your car radio: to get from 98.7 FM to 101.5 FM, you have to move to the right. The mental timeline has an especially pervasive influence. Beyond constraining the layout of printed timelines and wall calendars, the mental timeline predicts the arrangement of digital interfaces that range from the Start menu (on the left of a computer screen) to the Play icon on any media player (pointing right) and the Back button built into your smartphone (pointing left). This space–time mapping shows up in the analogue environment as well. For example, on store shelves in the US, shampoo bottles tend to be placed to the left of conditioner bottles, reflecting the order in which customers typically use these products (Figure 2A).

If the left-to-right mappings of time and number were common to all users, then designers could rely on their intuitions to develop interfaces that would be congenial to all users. However, the mental timeline and mental number line, like many other mental metaphors, are not universal. Rather, the specifics of people’s implicit spatial metaphors (i.e., their spatial directions and dimensions) vary across individuals and groups, as we discuss below. For this reason, following one’s intuitions about spatial metaphors can be misleading, causing designers to rely on metaphors that may be appropriate for their own demographic but at odds with the spatial metaphors in the minds of many other users.

## Cultural variation in spatial metaphors

However, natural it may seem for time to flow to the right and for numbers to increase to the right, these space–time and space-number mappings are culture-specific. In some non-Western cultures, the mental timeline and/or mental number line go in the opposite direction, from right to left (e.g., Tversky et al., 1991; Shaki et al., 2009; Fuhrman and Boroditsky, 2010). For people in those cultures, placing tomorrow to the right of today might seem obviously wrong – tomorrow belongs on the left! The culture-specific direction of the mental timeline is even reflected in the typical arrangement of consumer products; whereas shampoo bottles tend to be found on the left of conditioner bottles in the US (Figure 2A), these products tend to be arranged in the opposite way on store shelves in Israel, consistent with Israelis’ right-to-left mental timelines (Figure 2B).

Such manifestations of culture-specific spatial metaphors can be self-reinforcing. For example, given a mental timeline that goes from right to left, Hebrew speakers tend to arrange the built environment accordingly, which may be manifest in innumerable ways (beyond shampoo bottles), from the layout of interior spaces (Scharine and McBeath, 2002) to the way the dishes get done (i.e., dirty dishes on the right of the sink, clean on the left). These aspects of the built environment then provide additional evidence that earlier in time corresponds to right and later corresponds to left, reinforcing the same spatial mapping that shaped them.

Given these right-to-left directed mappings, many of the design elements that cater to Westerners’ implicit space–time and space-number metaphors are completely backwards for people in these other cultures, producing a potential conflict between the way these domains are spatialized in their minds and the way they are spatialized in the built environment.^2^ This conflict can have culture-specific effects on people’s preferences. For example, participants with rightward-directed mental timelines (e.g., native speakers of German, Italian, French, or English) prefer scenes depicting rightward movement (Chokron and De Agostini, 2000), images in which the agent (e.g., the person who’s swatting) is on the left side and the patient (e.g., the person who’s being swatted) is on the right (Maass et al., 2014; Esaulova et al., 2021), and arrays with older products (e.g., a typewriter) on the left and newer products (e.g., a computer) on the right (Zhang et al., 2022) compared to the opposite orientation. Strikingly, this lateral directional preference for visual stimuli disappears or reverses in participants whose reading habits – and therefore mental timelines – are mixed or fully right-to-left (e.g., native speakers of Farsi, Arabic, or Hebrew).

This conflict can also have culture-specific effects on learning and memory. For example, when asked to memorize arbitrary pairings of letters and shapes, English-speakers learned the pairings better when they were presented from left to right, congruent with the direction of their mental timelines, than when they were presented in the other direction. Hebrew-speakers showed the opposite culture-specific effect of direction; whereas right-to-left presentation impaired learning in English-speakers, it improved learning in Hebrew-speakers (McCrink and Shaki, 2016). Such findings illustrate why it is important that designers do not simply rely on their own intuitions even when laying out a simple user interface; what is natural and helpful to one group of users can be unnatural and detrimental to another.

Whereas the mental timeline and mental number line differ in spatial direction (e.g., rightward, leftward), other mental metaphors differ in their spatial dimensions (e.g., length, thickness, size). For example, to describe musical pitches, speakers of many languages including English and Dutch use unidimensional space; “high” pitches are associated with higher points in space and “low” pitches are with lower points in space. By contrast, in languages like Farsi, pitches are described as “thin” or “thick.” Speakers of “height languages” and “thickness languages” conceptualize pitch accordingly, as shown by non-linguistic tests of pitch reproduction. Whereas English and Dutch speakers implicitly associate high-and low-frequency pitches with high and low locations in space, Farsi speakers implicitly associate high-frequency pitches with thinner objects and low-frequency pitches with thicker objects (Dolscheid et al., 2013). This thickness-pitch mapping differs from the height-pitch mapping not in direction, but rather in the spatial dimension that serves as source domain (i.e., unidimensional height v. multidimensional thickness).

Similarly, different language groups use different spatial dimensions to conceptualize temporal duration, as well. English speakers conceptualize temporal duration using spatial length, as reflected in their linguistic metaphors for duration (e.g., a “short” or “long” vacation; Casasanto and Boroditsky, 2008). By contrast, Greek speakers conceptualize duration in multi-dimensional space – that is, volume rather than length (Casasanto, 2010). For Greek speakers, a long night is “big” in time, and a short night is “small,” a pattern found both in their linguistic metaphors and in their mental metaphors for duration (Casasanto et al., 2010).

In sum, spatial metaphors differ across cultures in a variety of ways: The mental timeline and mental number line differ in which direction they go, whereas the space-pitch and space–time metaphors described above differ in which spatial dimensions they use (i.e., height vs. thickness for pitch, length vs. volume for duration). Designing for these culture-specific mental metaphors cannot be accomplished by simple translation (Marcus, 2001). Even if an interface uses the appropriate linguistic metaphors, it can still violate its users’ mental metaphors by using the wrong spatial direction or spatial dimensionality. For example, at the bottom of every YouTube video is a red line that grows rightward as the video plays, which conforms to English-speakers’ conceptions of time as (a) linear and (b) rightward. For Arabic-speaking users, this user interface element could be flipped to accommodate the direction of their mental timeline (which progresses from right to left). However, no simple transformation of that line could accommodate Greek speakers’ preferred multi-dimensional conception of duration (i.e., as spatial volume); lines simply do not use the right kind of space. Although any one such metaphoric mismatch is unlikely to have serious consequences on its own, each one adds friction to the interaction and therefore subtly degrades the user experience.

## Individual variation in spatial metaphors

Mental metaphors not only vary across cultures; some also vary across individuals within a culture. A prime example of such variation is found in the lateral space-valence metaphor discussed above. The GOOD IS RIGHT mapping we focused on is found selectively in right-handed people, whose manual interactions with the environment are more fluent on average on the right side of space than on the left. Since people evaluate fluent experiences more positively than disfluent experiences, right-handers come to associate the right side of space with more positive emotions: a GOOD IS RIGHT mapping. Left-handers have the opposite pattern of manual experience (i.e., they tend to be more fluent on the left side of space) and they show the opposite set of associations between emotional valence and lateral space: an implicit GOOD IS LEFT mapping. In this way, lateral space-valence metaphors are *body-specific* (Casasanto, 2011); people with systematically different bodily experiences (e.g., as determined by their handedness) develop systematically different mental metaphors as a consequence (Casasanto, 2009; Casasanto and Chrysikou, 2011), with implications beyond the laboratory.

Whereas right-handers prefer objects on the right and disprefer objects on the left, the opposite is true for left-handers who, on average, prefer people, products, and pictures they encounter on the left side of a page or a screen (Casasanto et al., 2014, for a review). Likewise, whereas right-handers’ memories for the locations of positive events are biased to the right, left-handers’ memories for the same events are biased to the left (Brunyé et al., 2012). This body-specific space-valence mapping is also evident in the spontaneous gestures people produce while they speak. In the final debates of the 2004 and 2008 U.S. presidential elections, right-hand gestures tended to accompany more positive speech than left-hand gestures in the two right-handed candidates (Bush and Kerry), but the opposite pattern was found in the two left-handed candidates (McCain and Obama), who tended to gesture with their left hands when talking about more positive topics (Casasanto and Jasmin, 2010). The association of good and bad with one’s dominant and non-dominant sides of space is found in children as young as five years old (Casasanto and Henetz, 2012), suggesting that this implicit mental metaphor may be relevant to the design of educational materials and practices.

The left–right mapping of emotional valence has broad implications for UIs since many UIs require people to choose among options arrayed in space. In voting booths, for example, people typically encounter arrays of options, either as levers on a panel or as names on a printed ballot. As these options are often arranged in columns, some candidate’s names are found above or to the right of other candidate’s names. It has long been recognized that being listed at the top of a ballot confers a substantial advantage on candidates (Miller and Krosnick, 1998), but the reason for this advantage has remained unclear: In principle, this advantage could be due to temporal and/or numerical primacy, or to ballot-specific conventions such a listing the incumbent first. One study, however, suggests implicit spatial metaphors are at least partly responsible for ballot-order effects. In a stratified national sample, Americans were asked to vote in a simulated election, responding on ballots with candidates’ names arranged either vertically (i.e., above and below each other) or laterally (i.e., to the left and right of each other; Kim et al., 2015). In addition to showing a trend toward the typical top-of-ballot advantage, the results showed that the left–right positions of candidates’ names affected how many votes they received. Left-handers were 15 percentage points more likely than right-handers to select the candidate whose name was on the left of the ballot, a body-specific effect of people’s implicit space-valence metaphors. Such findings highlight a challenge for designers: Incidental spatial relationships in UIs can have unintended (and potentially serious) consequences, systematically biasing some users toward one set of choices and other users toward the alternative choices.

**Figure 3:**
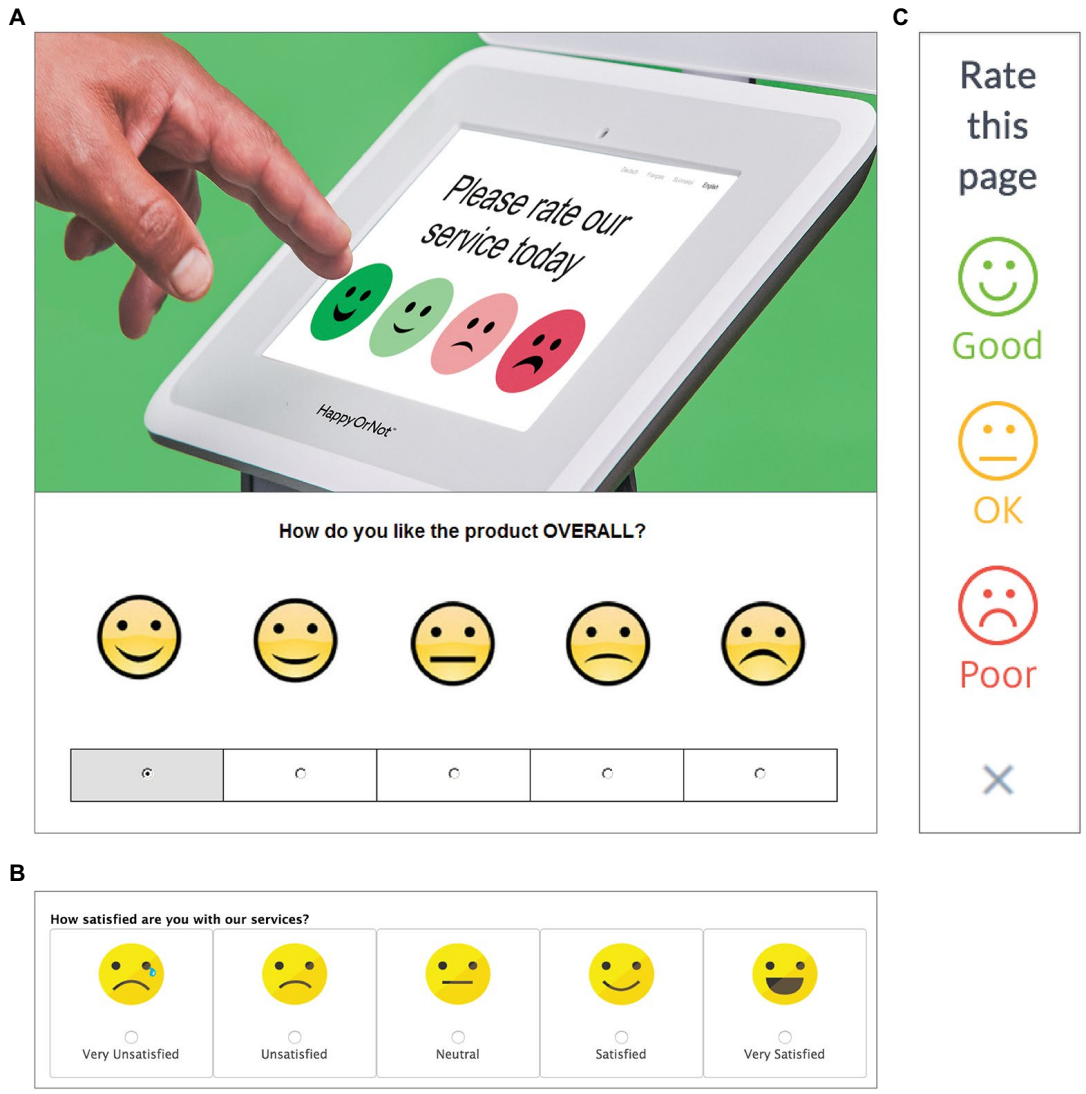
The left–right spatialization of emotions on rating scales often violates the GOOD IS RIGHT mapping characteristic of most people (i.e., right-handers). **(A)** Interfaces that are congruent with that mapping **(B)**, are necessarily incongruent with the GOOD IS LEFT mapping of left-handers. Vertical rating scales **(C)** leverage the universal GOOD IS UP mapping shared by all users. Reproduced with permission from Happy or Not, available at www.happy-or-not.com.

Fortunately, as researchers learn more about mental metaphors, designers can be better equipped to anticipate many of the spatial biases that people exhibit. With this knowledge, designers can not only mitigate the unintended effects of those biases (e.g., by randomizing the location of candidate names on ballots, a practice that has not been universally adopted across United States voting districts), but can also use implicit spatial metaphors strategically to optimize user experience. For example, when eliciting ratings from users of a website, a restaurant, or an airport bathroom, companies often use a rating scale consisting of smiley and frowny faces arranged in lateral space (see Figure 3A), similar to the emotion scales used by teachers and mental health professionals. In many cases, these faces are presented with the happiest faces on the left and the saddest faces on the right, an arrangement that is directly in conflict with the GOOD IS RIGHT mapping held by 90% of the population (i.e., right-handed people).^3^ As a result, for most people, the most common interface for rating one’s experience is backward, presenting happy faces on the negative side of space and sad faces on the positive side. When the faces are presented in the other direction, as they are in some cases (Figure 3B), this arrangement is consistent with the GOOD IS RIGHT mapping of most users but is inconsistent with the GOOD IS LEFT mapping of a substantial minority: left-handed users (e.g., over 30 million Americans). Whichever way the faces are arrayed in left–right space, such interfaces contradict the lateral space-valence metaphor of some subset of users, and this conflict likely introduces a source of noise into ratings data; some of the most satisfied users may inadvertently choose the saddest face (because it is located on their “good” side) and some of the least satisfied users may choose the happiest face (located on their “bad” side).

How can designers accommodate such individual variation in mental metaphors? In the case of rating scales, no set of laterally-presented smiley faces could be congruent with the lateral space-valence mappings of both left-handed and right-handed users, since their mappings go in opposite directions. Rather, the solution may simply be to orient the smiley faces in vertical space, as some interfaces already do (Figure 3C). Given that the GOOD IS UP metaphor appears to be nearly universal (and does not vary with handedness or writing direction; Casasanto, 2009; Zhang et al., 2022; *cf*., Wnuk and Ito, 2021) it provides a single arrangement of faces that is congruent with a space-valence metaphor that is presumably found in all users’ minds. Although there is a simple design solution in the case of rating scales, many other UI elements do not have a one-size-fits-all solution. Rather, in order to fully leverage the latent structure of people’s minds, an interface must be language-specific, culture-specific, or body-specific — like the mental metaphors of its users. In the next section, we discuss a principled way to predict how mental metaphors vary across individuals and groups.

## How to anticipate people’s spatial metaphors

Spatial metaphors can be spoken or unspoken, and they can vary widely across cultures and individuals. Nevertheless, researchers and designers alike can make clear predictions about the structure of mental metaphors (i.e., source-target mappings in the mind) using the CORrelations in Experience (CORE) principle (Pitt and Casasanto, 2020). According to the CORE principle, the way source domains (like space) and target domains (like musical pitch or emotional valence) are mapped in the mind is determined by the way those domains are correlated in a person’s experience. When applied to spatial metaphors (i.e., mental metaphors in which the source domain in space), the principle simplifies to: Abstract domains are spatialized in the mind according to the way they are spatialized in experience. This simple principle allows us to use the structure of people’s experience to make predictions about the structure of their mental metaphors. The CORE principle can explain all of the mental metaphors discussed above and why they vary in the ways they do. Three examples show how mental metaphors are predictable on the basis of people’s cultural, linguistic, and bodily practices.

### Predicting space–time metaphors from cultural practices

The direction of the mental timeline has been attributed to the direction of reading and writing across cultures (e.g., Tversky et al., 1991; Fuhrman and Boroditsky, 2010; Casasanto and Bottini, 2014b) but why would reading experience shape the way people think about time? When reading a line of English text, the reader’s gaze starts on the left side of the page at an earlier time and ends on the right side of the page at a later time. This experience provides a correlation between space and time in the act of reading, and this space–time correlation is reversed when reading Hebrew or Arabic text (which is written from right to left). Therefore, according to the CORE principle, reading text left-to-right should yield a mental timeline that progresses rightward, and reading text right-to-left should yield a mental timeline that progresses leftward, consistent with the variation in mental timelines observed across cultures.

The relationship between the direction of reading and writing and the direction of the mental timeline is more than just correlational. In a series of training studies (Casasanto and Bottini, 2014b; Pitt and Casasanto, 2020), participants read the same text either normally (left-to-right) or in mirror-reversed orthography (right-to-left) and then performed a test of their implicit mental timelines (based on reaction times). This laboratory manipulation tests whether reading experience can cause changes to the mental timeline, holding other variables constant. And it can; just a few minutes of reading mirror-reversed text is enough to weaken or even reverse people’s normal mental timelines.

### Predicting space-valence metaphors from bodily practices

Why do right-handers associate right with positive emotions and left with negative emotions, and why do these associations reverse in left-handers? Although the form of the human body is largely symmetrical across left–right space, the way we use our bodies is lopsided; most people have a dominant hand with which they can interact with the world more easily than with the nondominant hand. Right-handers can interact with their environment more fluently on the right side of space, whereas left-handers experience more fluent body-world interactions on the left. A large literature has established links between fluency and positive evaluations: People tend to like things better when they experience them more fluently (Reber et al., 2004). Therefore, the asymmetry in manual fluency across people’s two hands provides a correlation in bodily experience between side of space and emotional valence; the dominant-hand side is fluent (i.e., positive), the non-dominant side is relatively disfluent (i.e., negative). On the basis of this bodily experience, the CORE principle predicts the body-specific space-valence metaphors that are observed: GOOD IS RIGHT for right-handers and GOOD IS LEFT for left-handers (Casasanto, 2009). If this body-specific pattern results from differences in manual fluency (and not from differences in the hemispheric lateralization of emotional valence, for example), then changing people’s manual fluency should cause changes to their lateral space-valence mappings. Consistent with CORE, transiently impairing the manual fluency of people’s dominant hand (by putting a bulky ski glove on it) reversed their normal space-valence mappings (Casasanto and Chrysikou, 2011). Changing the way people use their hands to interact with their environment changes the correlations they experience between space and valence, dynamically shaping their feelings and evaluations.

### Predicting space-pitch metaphors from linguistic practices

Why do different cultures have different mental metaphors for musical pitch? In this case, the answer is found in language. When people describe pitches as “high” or “low” this metaphor in language can cause the listener to activate mental representations of vertical space – concepts of literal high or low spatial position. Likewise, when people describe pitches as “thick” or “thin,” as Farsi speakers do, this metaphor in language can cause the listener to activate mental representations of spatial thickness. These different linguistic metaphors provide different correlations between representations of pitch and space. Given that these different linguistic metaphors provide different correlations between linguistic and perceptual experiences of pitch, CORE predicts the difference in mental metaphors observed in Dutch and Farsi speakers discussed above (Dolscheid et al., 2013). Beyond this correlation, CORE also predicts that using a new linguistic metaphor for pitch should cause people to adopt a different mental metaphor. Indeed, training speakers of a “height” language to describe pitches in terms of thickness (e.g., “A tuba sounds thicker than a flute”) made them sensitive to the thickness of a task-irrelevant spatial stimulus; they estimated the pitches of target notes to be higher after seeing thinner lines on a computer screen and to be lower after seeing thicker lines on the screen, just like native speakers of a “thickness” language (Dolscheid et al., 2013).

### Predicting possible mappings

As the previous examples illustrate, spatial mappings are remarkably flexible, changing or reversing in response to long-term changes in an individual’s language, culture, or body, and even changing in response to brief laboratory interventions. How could such spatial mappings of non-spatial concepts be so flexible, while also providing a reliable basis for conceptual structure? And what are the limits of this flexibility?

An answer to both of these questions is given by Hierarchical Mental Metaphor Theory (HMMT; Casasanto and Bottini, 2014a; Casasanto, 2017), the parent theory of the CORE principle. According to HMMT, the CORE principle operates on multiple timescales, over different sets of experiences. Each specific spatial mapping that a person exhibits is a member of a larger family of mappings, all of which are present in the same mind. For example, although English-speakers show strong left-to-right mental timelines, this is not the only spatial mapping of temporal order that they have in their minds; rather, they have a family of mappings that link progress through time to progress through space in any direction (i.e., leftward, downward, away, etc.) The reason that many people show a specific mapping (rather than omni-direction mappings) is not because they lack other mappings but rather because the most recent and frequent correlations they have experienced (i.e., *via* their culture, language, and/or body) favor one mapping over the others. For example, reading text from left to right provides a correlation between progress through time and progress rightward through space, and therefore strengthens the rightward-directed mapping of time at the expense of the other mappings in the same family. This structural feature of mental metaphors explains their surprising flexibility; new experiences (e.g., the direction of written text, the relative fluency of the hands, or patterns in metaphorical language) neither create new mappings nor destroy old mappings. Rather, these experiences transiently shift the weight of evidence from one mapping to another within a pre-existing family of competing mappings, strengthening some and weakening others. In this way, the structure of mental metaphors is stable at one level (i.e., a family of mappings) and flexible at another level (i.e., specific mappings).

Importantly, the flexibility of spatial metaphors is not without limits. According to HMMT, all of the correlations that people experience between a given target domain (e.g., duration) and various spatial source domains (e.g., length, volume) are members of the same family. Whereas the relative strengths of specific mappings are determined by correlations in cultural, linguistic, or bodily experience, the superordinate families of mappings typically arise from correlations the source and target domains in the natural world. For example, as objects travel farther through space more time passes, providing a correlation in experience (CORE) between duration and distance that is observable in the motion of any object. Likewise, as substances accumulate in a container (e.g., water filling a bottle) more time passes, providing a correlation in experience between duration and volume. These correlations hold for anyone interacting with the natural world, resulting in a family of space–time mappings that are presumably shared by all people: a universal basis for the culture-specific, language-specific, and body-specific mappings observed across groups (Pitt et al., 2021). In this way, the set of spatial mappings that people can flexibly use for a given target domain is constrained by the COREs that are observable in the natural world.

This prediction of HMMT has been borne out by the limited flexibility of space-pitch metaphors. In the natural world, thicker objects tend to make lower pitches; big drums make lower-pitch sounds than small drums, big animals make lower-pitch sounds than small animals, and thick strings make lower-pitch sounds than thin strings when plucked. According to HMMT, this thickness-pitch mapping (i.e., thicker ~ lower) should be latent in the minds of all people, but that the opposite mapping (i.e., thicker ~ higher) should not. Studies of both pre-linguistic infants and of adults speakers of a height-pitch language (like English) were sensitive to the thickness-pitch relationship that is prevalent in the natural world, but not to the opposite thickness-pitch relationship (Dolscheid et al., 2013, 2014). These findings suggest that although people are exquisitely sensitive to changes in the correlational structure of their environment, their spatial metaphors are only so flexible, bounded by the set of source-target correlations that are observable in the natural world.

## Seven opportunities and challenges for designers

1. **Make space for thinking**. Space serves as the source domain in a wide variety of mental metaphors, supporting reasoning about time, numbers, pitch, emotional valence, and similarity, among others. Given the central role that space plays in representing so many basic concepts, it is important that interfaces give users enough space to think. As our digital devices become ever smaller, with interfaces as small as a coin, designers must innovate ways to use a shrinking amount of screen real estate to communicate increasingly sophisticated information.
2. **Spatialize with care**. To use space effectively, designers should leverage the spatial metaphors that their users bring to the UI. When something is spatialized (e.g., on screen), it is likely that it either conforms or conflicts with one or more implicit mappings in the user’s mind. Interactions that conflict with a user’s mappings will be less fluent, less pleasant, and less effective. Interactions that leverage these mappings can improve users’ speed, memory, and mood.
3. **Get out of your own head**. Do not assume that the spatial metaphors that feel natural to you will feel natural to your users. Different people use different implicit spatial metaphors.
4. **Take your cues from COREs**. To anticipate which spatial metaphors a person is likely to prefer, look beyond the metaphors in their language to the correlations in their non-linguistic cultural and bodily experiences as well. In addition to the insights from psychological experiments, design researchers can incorporate ethnographic methods (e.g., structured interviews, observational studies, and artifact analysis; Blomberg and Burrel, 2009) for predicting the spatial metaphors in target groups: Observing the behaviors, objects, and environments that shape people’s everyday experiences can provide insight about the spatial metaphors that likely shape their minds, even when they are absent from language.
5. **One size fits some**. When possible, adopt a design solution that accords with universal spatial metaphors (e.g., GOOD IS UP). In many cases, no one design solution will accord with the spatial metaphors of all users. In these cases, ask: (a) How do they talk about this thing? (b) How is it laid out in the cultural artifacts and practices they already use? (c) Do people with different bodies experience it in systematically different ways?
6. **Minds are malleable so handle with care**. Given that mental metaphors can be changed by experience, designers who determine the structure people’s everyday experiences inadvertently become stewards of their mental metaphors. Any experience that spatializes an abstract idea has the potential either to reinforce the implicit spatial metaphor that structures that idea or to systematically change it. Designers, therefore, should be aware of their power not only to leverage, but also to alter, the metaphorical structure of users’ minds.
7. **Respect the natural boundaries of spatial metaphors**. Although spatial metaphors can shift in response to experience, they are not infinitely flexible. Rather, correlations in the natural world define the set of mappings that is available to users. Any spatial metaphor should respect these universal boundaries.

## Conclusion

Many of our most basic concepts are structured by spatial metaphors, including concepts of time, number, musical pitch, and emotional valence. Whether or not these metaphors are found in language, they have pervasive and sometimes profound effects on the way people think, feel, and act. Spatial metaphors are so pervasive that nearly any user interface will inevitably engage with at least one of them, whether intentionally or unintentionally (after all, spatializing non-spatial ideas is what graphical UIs do!). The goal of user experience designers should be not only to make interfaces intuitive and inclusive (Hurtienne et al., 2008), but also to make user experiences that help people think, feel, and act more efficiently. User experiences that conform to people’s mental metaphors can improve learning and increase positive evaluations, whereas experiences that violate their mental metaphors can impair learning and depress evaluations.

The biggest challenge may be to design interfaces that accommodate (or even better, leverage) the multi-dimensional variation in people’s mental metaphors. How can designers anticipate the mental metaphors of their users when those metaphors vary within and across cultures, and are often nowhere to be found in language? According to the Correlations in Experience principle, the answer lies in people’s everyday experiences, whether those experiences are cultural, linguistic, or bodily. CORE provides a way for researchers and designers alike to make principled predictions about the structure of people’s minds from the structure of their experiences, and gives the people who shape our physical and digital environments a comprehensive, user-centered set of metaphors to design by.

## Author contributions

BP wrote the manuscript with guidance and editing from DC. All authors contributed to the article and approved the submitted version.

## Conflict of interest

The authors declare that the research was conducted in the absence of any commercial or financial relationships that could be construed as a potential conflict of interest.

## Publisher’s note

All claims expressed in this article are solely those of the authors and do not necessarily represent those of their affiliated organizations, or those of the publisher, the editors and the reviewers. Any product that may be evaluated in this article, or claim that may be made by its manufacturer, is not guaranteed or endorsed by the publisher.

## Footnotes

^1^Although the GOOD IS RIGHT metaphor obtains for right-handers (approximately 90% of the population), left-handers show the reverse metaphor. We discuss this topic in depth below.

^2^In an effort to minimize such conflicts in spatial metaphors, a subset of user interfaces are designed to support both right-to-left and left-to-right layouts. For example, when an Israeli opens Microsoft Excel, the first cell that is highlighted is the upper right cell, not the upper left. But these right-to-left layouts are rare and often incomplete, leaving entire populations to live with the difficulty that comes from mixing metaphors.

^3^On what basis are these faces arranged in Figure 3A, if not according to a GOOD IS RIGHT mapping? Presenting happier faces on the left and sadder faces in the right likely reflects the order in which the words "happy" and "sad" are typically spoken and written; following a principle known as linguistic markedness (Clark, 1973), people are more likely to say "happy or sad" than "sad or happy."
